# A tissue biopsy-based epigenetic multiplex PCR assay for prostate cancer detection

**DOI:** 10.1186/1471-2490-12-16

**Published:** 2012-06-06

**Authors:** Leander Van Neste, Joseph Bigley, Adam Toll, Gaëtan Otto, James Clark, Paul Delrée, Wim Van Criekinge, Jonathan I Epstein

**Affiliations:** 1 MDxHealth, Irvine, CA, USA; 2 James Buchanan Brady Urological Institute, The Johns Hopkins University School of Medicine, Baltimore, MD, USA; 3 Institut de Pathologie Génétique, Gosselies, Belgium

**Keywords:** GSTP1, APC, RASSF1, Methylation, Epigenetics, Prostate cancer, Diagnosis, Multiplex, Singleplex, MSP

## Abstract

**Background:**

PSA-directed prostate cancer screening leads to a high rate of false positive identifications and an unnecessary biopsy burden. Epigenetic biomarkers have proven useful, exhibiting frequent and abundant inactivation of tumor suppressor genes through such mechanisms. An epigenetic, multiplex PCR test for prostate cancer diagnosis could provide physicians with better tools to help their patients. Biomarkers like *GSTP1*, *APC* and *RASSF1* have demonstrated involvement with prostate cancer, with the latter two genes playing prominent roles in the field effect. The epigenetic states of these genes can be used to assess the likelihood of cancer presence or absence.

**Results:**

An initial test cohort of 30 prostate cancer-positive samples and 12 cancer-negative samples was used as basis for the development and optimization of an epigenetic multiplex assay based on the *GSTP1*, *APC* and *RASSF1* genes, using methylation specific PCR (MSP). The effect of prostate needle core biopsy sample volume and age of formalin-fixed paraffin-embedded (FFPE) samples was evaluated on an independent follow-up cohort of 51 cancer-positive patients. Multiplexing affects copy number calculations in a consistent way per assay. Methylation ratios are therefore altered compared to the respective singleplex assays, but the correlation with patient outcome remains equivalent. In addition, tissue-biopsy samples as small as 20 μm can be used to detect methylation in a reliable manner. The age of FFPE-samples does have a negative impact on DNA quality and quantity.

**Conclusions:**

The developed multiplex assay appears functionally similar to individual singleplex assays, with the benefit of lower tissue requirements, lower cost and decreased signal variation. This assay can be applied to small biopsy specimens, down to 20 microns, widening clinical applicability. Increasing the sample volume can compensate the loss of DNA quality and quantity in older samples.

## Background

In the USA, over 70% of the annually ~1,000,000 performed biopsies for prostate cancer suspicion result in a cancer-free diagnosis [1-4], with screening programs typically based on serum PSA levels [5,6]. Due to the limited performance of this marker in terms of suboptimal sensitivity and specificity, there have been recommendations against the utilization of serum PSA levels for prostate cancer screening [7-9]. Several alternatives are actively being explored for prostate cancer screening and detection. This report emphasizes one particular biomarker strategy,i.e. DNA methylation of key gene promoter regions. DNA hypermethylation is an epigenetic change that locks genes in a silent, non-expressed state [10]. Such changes occur both during normal development and during oncogenic processes [11,12]. In cancer, DNA methylation is both an abundant and frequent event, leading to the functional loss of key tumor suppressor genes [13]. Through the progressive accumulation of aberrant methylation during oncogenesis, epigenetic changes can occur in precancerous lesions or at some distance of the actual tumor focus, a phenomenon termed field effect [14]. The latter could be an important advantage to overcome suboptimal biopsy localization and false negative findings due to tissue core sampling errors [15,16].

From the diagnostic perspective, creating a robust, accurate assay is of paramount importance. In addition, from the clinical point of view, the assay should be able to test readily available samples with a range of quantity and quality. When a diagnostic assay consists of several subtests, e.g. a panel of several biomarkers, these can, potentially, be run together in a multiplexed fashion. Although a multiplex assay minimizes sample volume requirements and specimen handling, and the associated operator-related and technical errors, it is important to verify that the generated output reaches the same standards of accuracy and reliability, compared to the individual singleplex biomarker versions.

The current study evaluates a multiplex assay for prostate cancer detection. To reduce the number of false positives and subsequent unnecessary (repeat) biopsies due to a lack of PSA-specificity and spurious extraction of biopsies cores [15-17], a confirmatory, epigenetic test can be run on previously collected prostate tissue biopsies [2,18]. The available samples for epigenetic testing are typically formalin-fixed paraffin-embedded (FFPE) samples used for histopathological analysis and then routinely archived. The methylation status of a panel of genes associated with prostate cancer can be determined on a few sections of such archived tissue blocks. In the present report, a diagnostic tissue-based test for prostate cancer presence consists of the epigenetic statuses of 3 genes, i.e. *GSTP1APC* and *RASSF1*[19], in addition to one control gene, *ACTB*, used to quantitate amplifiable DNA.

## Results and discussion

### Multiplexing affects copy numbers

Thirty cancer positive tissue samples and 12 controls from cancer-free individuals were analyzed with 4 singleplex assays versus 1 multiplex assay. Samples are typically split into 2 equal aliquots to assure output. The control gene *ACTB* was used as a rough estimate of input DNA quantity and quality. The ratio of the *ACTB* copies in both replicates ranged from .73 to 1.17 (one outlier of 1.42 removed) for the multiplex assay and from .74 to 1.19 for the singleplex assay. The medians of these ratios were close to 1 for both the singleplex and multiplex assays, i.e. 1.01 and 1 respectively, as could typically be expected for a distribution of intra-run noise. Moreover, the 2 equal aliquots generated similar output in terms of *ACTB* copy numbers for both the singleplex and multiplex assays separately (p-value = .33 & .24 for the multiplex and singleplex assay respectively; Mann–Whitney paired sample test). Hence, copy numbers from both aliquots were averaged in all subsequent analyses.

**Figure 1:**
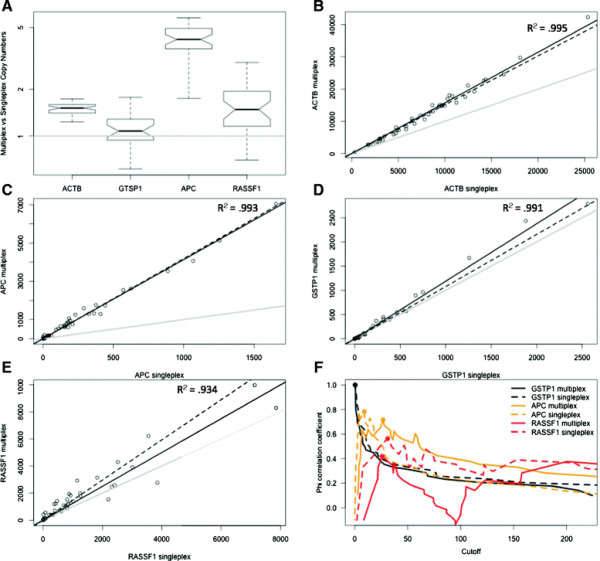
**Output characteristics of the all-in-one multiplex assay versus 4 singleplex assays.** (**A**) Relative copy number changes for the individual assays for paired samples presented as the ratio of the multiplex over the singleplex copy numbers. Dot plots for the paired copy numbers of *ACTB* (**B**), *APC* (**C**), *GSTP1* (**D**) and *RASSF1* (**E**) for the multiplex assay (y-axis) versus the singleplex assay (x-axis). The gray line represents identity, i.e. equal result for both versions of the assay, while the black, dashed line represents the median signal change obtained from the transition of a singleplex to a multiplex assay. The greater the angle between both lines, the higher the increase. The results from the linear fit are shown with the black, full line. The quality of the fit is represented by the adjusted R^2^-values. (**F**) Phi or Matthews correlation coefficient (MCC) in function of the methylation ratio that is used as a cutoff. The maximal value for MCC is shown as a circle, for each individual assay. Shifts due to the transition of singleplex to multiplex, between the optimal cutoffs, identified here through maximal correlation, can be observed for each assay separately.

*ACTB* copy numbers were significantly higher for the multiplex assay compared to the singleplex assay (p-value = 4.5e-13; Mann–Whitney paired sample test), exhibiting a median 1.51-fold copy number increase (range: 1.23–1.73; Figure 1A & B). Similarly, this effect was very pronounced for the *APC* methylation specific assay (Figure 1A & C), with a median copy number increase of 4.19-fold over the singleplex (p-value = 2.7e-08), while the same effect was less conspicuous for *RASSF1* (p-value = 3.0e-06) and only marginally visible for *GSTP1* (p-value = .00705), which exhibited a median increase of 1.48-fold and 1.08-fold for matched samples, respectively (Figure 1A, D & E).

Finally, by fitting a linear model, the copy number changes introduced through assay multiplexing can be predicted. Because no template controls yielded 0 copies for all multiplex and singleplex assays, the model was generated including the origin. In line with the median increases, the linear regression yielded amplification factors of 1.57, 1.19, 4.13 and 1.25 for the *ACTB*, *GSTP1*, *APC* and *RASSF1* assays respectively (Figures 1B–1E).

In conclusion, multiplexing three methylation specific assays together with the *ACTB* control assay resulted in differences in the individual analytical readouts, without affecting the stability, as indicated by the consistently high R-square values for the linear models (Figure 1B–E). These changes were most likely linked through the different sensitivity of the fluorophores that are used to separate the signals of the individual assays. This is best evidenced by the less pronounced copy number changes for *GSTP1*, which is assessed with the same fluorophore in both the singleplex and the multiplex setup (Additional file 1: Table S1). Therefore, analytical cutoffs for methylation levels will need to be determined independently for singleplex and multiplex assays.

### Methylation test performance indications

Q-MSP methylation results are most often reported as a ratio of methylated copies of the target gene divided by the copy number of a control gene, typically *ACTB*, and multiplied by 1000 for convenience. As expected from the copy number changes occurring due to multiplexing, the ratios of the multiplexed *GSTP1* assay were significantly lower than the singleplex version (p-value = 3.3e-06; Mann–Whitney paired sample test), while the reverse was true for *APC* (p-value = 2.9e-08). Since the effect of multiplexing on the copy numbers of both *RASSF1* and *ACTB* was fairly similar, the *RASSF1* ratios were not altered significantly (p = .96).

In this limited assay development cohort, consisting of actual tumor samples and non-cancerous controls, methylated *GSTP1* copies were observed in all cases, but none of the controls, leading towards a perfect classification and test performance for both the multiplex and singleplex assays (Figure 1F, Additional file 1: Figure S1). As could be expected, multiplexing the *APC* assay introduced an upward shift of the optimal *APC* cutoff ratio to separate cases from controls, in line with the general trends for copy numbers and methylation ratios. The optimal cutoff ratio remained similar, although with a slight decrease, for the multiplexed *RASSF1* assay. Due to the predictability and stability of these events, the small decrease of Phi, or Matthews correlation coefficient (MCC), a preferred substitute over test accuracy for small, unbalanced populations [20], is most likely due to random events, rather than being a consistent error introduced through multiplexing. Indeed, the maximum Phi obtained equals .72 for the multiplex *APC* versus .78 for the singleplex *APC* assay, and .42 versus .56 for the multiplex and singleplex *RASSF1* assays (Figure 1F). ROC-curves (Additional file 1: Figure S1) for all the assays confirm these trends, although these are only intended for indicative purposes only due to the small sample size and inadequate constitution of this assay development cohort.

In conclusion, multiplexing the *GSTP1*, *APC* and *RASSF1* methylation assays does not appear to have an adverse effect on detecting cancer accurately, especially when all 3 assay’ results are considered as a whole. Multiplexing and the use of different fluorophores do affect the copy numbers of the individual assays, leading to altered ratios and classification cutoffs. Thus, analytical cutoffs for optimal sensitivity and specificity are only valid for either the singleplex or multiplex diagnostic assay. Although multiplexing did not change the final tissue sample outcome for *GSTP1*, the correlation between methylation status and outcome did slightly decrease for *APC* and *RASSF1*. Since there does seem to be a consistent trend in the way multiplexing affects a particular singleplex assay (Figure 1), the changes in MCC for *APC* and *RASSF1* are likely random events due to the small sample size of this cohort.

### Tissue minimization and sample age

Several reasons could warrant the transition of multiple diagnostic singleplex assays to one multiplex product. Minimizing the potential operator and technical errors has already been highlighted. The need for sufficient sample quantity is an additional factor that should be taken into account. In this light, Q-MSP results of the multiplex assay were tested in decreasing amounts of biopsy sections. To this end, biopsies consisting of 10, 20 and 40 μm from FFPE tissue blocks from the minimization cohort were tested and compared.

Two outliers, that yielded copy number values for *RASSF1* over 10-fold of the maximally obtained *ACTB* copy number, were removed from the analysis. For non-descriptive analyses, samples from 2005 and 2006 are grouped and considered old samples, i.e. 5 years or more since fixation and archiving. Samples from 2011 are considered new, i.e. recently fixated and archived.

**Figure 2:**
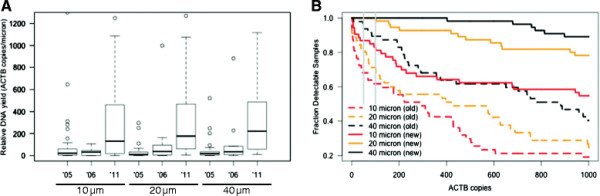
**Reliably measuring DNA input quality and quantity.** (**A**) Relative DNA yield in terms of *ACTB* copy numbers per micron of biopsy tissue, stratified by age and sample quantity groups. Outliers, i.e. data points exceeding 1.5 times the respective interquartile distances, are not represented. (**B**) Relative amount of detectable samples, in terms of possessing a minimum of *ACTB* copies, as a function of the total amount of *ACTB*. Gray horizontal lines represent possible, minimal cutoffs to assure sufficient DNA quantity and quality, set here at 50 and 100 *ACTB* copies.

A comparison of relative DNA quantity and quality can be made between all these biopsy samples taking into account the tissue volume and the sample age (Figure 2A). The effect of the sample age is quite obvious, with older samples from 2005 and 2006 clearly showing lower relative DNA yields, measured as *ACTB* copies per micron. The effect of the original sample volume on the relative DNA yield appears to be minor, although a small increase for larger volumes can be observed. The conditions for an analysis of variance are not strictly fulfilled, however, this test is used to further elucidate the relative contributions of sample age and sample volume. A linear model can be fit to the relative *ACTB* copy numbers, or the log of the relative *ACTB* copy numbers, since the latter fits the normality assumptions better. Both models indicate consistent results, with the age of samples being the only significant factor (p = 3.9e-11; ANOVA).

In conclusion, the age of samples has a significant impact on the DNA quantity and quality. Increased sample volume is likely to be beneficial to recover more DNA, which could be of noteworthy importance for conducting the assay on older samples. Although larger sample volumes do seem to result in a higher DNA extraction efficiency (Figure 2A), this does not appear to be significant.

### Sample usability and DNA detectability

With lower amounts of sample input and sample ageing, the likelihood increases that signals will be indistinguishable from noise and therefore lost. Assessing the status of the control gene first, 11 samples failed to show any *ACTB* copies in either one of the aliquots, of which 10 are classified as older samples (8 from 2005). In addition, 6 of these samples are from 10-micron specimens, 4 from 20-micron biopsies and only 1 from a 40-micron biopsy. Obviously, higher sample quantity and more recent sample fixation, minimize the chance of producing an undetectable *ACTB* signal. In addition, the majority of the undetectable signals occur in only one of the replicates. When the averaged aliquots are considered instead, only 5 older samples did not contain any *ACTB* copies, of which three were 20-micron samples and two were 10-micron samples. Figure 2B illustrates the fraction of detectable samples as a function of the *ACTB* copy number. All findings concerning trends for DNA yield relative to sample age and sample quantity are reinforced.

The minimal DNA quantity, in terms of *ACTB* copies, that results reliably, i.e. more than 0 copies for both replicates, in the detection of methylated copies of *GSTP1*, *APC* and *RASSF1* equals to 51, 27 and 63 respectively. A cutoff on *ACTB* of 50 to 100 copies should result in the reliable detection of methylation of all three genes. Below the cutoff of 63, not detecting methylated copies of either assay could be either due to a true lack of methylation or due to insufficient qualitative DNA. From Figure 2B it can be derived that for recent samples, 20-micron is sufficient, with no sample loss due to undetectable *ACTB* copies. For samples up to 5 years of age, a loss can be expected almost regardless of the tissue volume. Allowing a maximal sample loss of 20%, 40-micron samples pose no problem, while 20-micron samples become borderline usable.

### Effects of quantity and quality restrictions

Probably the most important characteristic of any clinical test is the final sample classification, i.e. whether methylated copies of a test gene can be detected in the case of an epigenetic assay. Key to this is the link between the amount of input material and the possibility of detecting methylation signals. For the tissue minimization study, the methylation ratio was determined using three, matched, biopsy-volume levels, i.e. 10, 20 and 40 microns. If methylation was observed in any of these, the biopsy was considered methylated. Methylation can be missed due to a lack of input material or due to the random sampling technique of the biopsy tissue relative to the tumor’s location within the prostate gland. The random combination of tissue block sections having variable methylation levels could also impact detection.

Samples were considered for subsequent analyses when the minimal DNA quantity and quality requirements were met, i.e. when *ACTB* exceeded 63 copies, as determined above. Table 1 lists the frequencies of accurately analyzable samples in terms of the ability to detect sufficient DNA and methylation signals, relative to the sample volume and age. More recent and larger volume samples clearly outperformed older, smaller ones. Especially the 10-micron samples suffered from insufficient input material in terms of *ACTB* copies. If sufficient DNA can be detected, the methylation signal was picked up reliably for each assay, down to a volume as low as 20 μm (Table 1).

**Table 1:** Relative sample amount for which methylation can be determined as function of quantity and age

**tissue biopsy volume**	**sample age**	**# samples (% correctly analyzable)**	**sufficient input DNA**	**methylated copies of**		
				**GSTP1**	**APC**	**RASSF1**
10 μm	> = 5 years	45 (60%)	67%	81%	81%	48%
	<= 1 year	53 (72%)	87%	80%	86%	84%
20 μm	> = 5 years	45 (73%)	78%	90%	93%	81%
	<= 1 year	53 (92%)	100%	91%	94%	93%
40 μm	> = 5 years	45 (82%)	93%	86%	88%	83%
	<= 1 year	53 (92%)	100%	96%	90%	93%

Samples eligible for downstream analyses have sufficient quantity and quality of input DNA. For methylation, only samples with sufficient DNA (over 63 *ACTB* copies) are considered. Samples are labeled methylated when exceeding .27, 26.8 or 25.8 copies for *GSTP1*, *APC* and *RASSF1* respectively, as preliminary determined based on the results of Figure 1F, optimizing for maximal correlation with the presence of cancer foci. The percentage of correctly analyzable samples is the combination of sufficient input DNA and detectability of methylation signals of either assay, when methylation should have been detected.

The loss of methylation signals in the 40-micron samples was most likely due to the randomness of the combination of tissue slices and the presence of cancer foci within the biopsy tissue tested in the assay. Indeed, methylation of either assay did not exceed the respective critical threshold for 4 of the recent, 40-micron samples, although results of the 10- or 20-micron samples indicated methylation. However, all four samples possess ample quantities of *ACTB*, i.e. with a minimum of 680 copies. Therefore, the lack of sufficient, high quality DNA was unlikely to explain the small amount of samples where methylation was not observed for any epigenetic assay, although at least one positive signal was expected.

Table 1 also lists the relative amount of samples that were accurately analyzable and gives the correct sample outcome as a function of age and volume. Such samples possessed a minimal amount of DNA that appeared to be sufficient for downstream Q-MSP analyses, and in these, methylation was detected reliably. Older, up to 5 years, 20-micron samples seem to be at the current technical limit for methylation analyses.

## Conclusions

Screening of prostate cancer suffers from a lack of specificity, identifying many false positives, mainly through the use of routine PSA screening and follow-up biopsy tissue analysis [21]. Men with elevated PSA-levels but negative biopsies will typically be re-evaluated and may be rebiopsied, although this has been proven unnecessary for the vast majority [22,23]. Epigenetics, more specifically DNA methylation, has been postulated to aid the diagnostic process. Because the presence of cancer cells missed by standard histopathology can be detected through DNA methylation [14], a confirmatory epigenetic assay can reinforce a negative finding or indicate the need for a rebiopsy in men with an initial negative biopsy [2].

DNA methylation plays a role in gene silencing and acts as a powerful biomarker by repressing tumor suppressor genes during oncogenesis [24]. The functional loss of key genes in normal cells is progressive in nature, accumulating deregulating oncogenic hits and eventually disturbing several protective regulatory pathways [25]. In multiple cancer types, including prostate cancer, this phenomenon is present as a field effect, offering the possibility to detect aberrant methylation at a distance from the actual tumor [14], and is currently used to develop an assay to improve upon standard histopathology in prostate cancer detection.

Several genes have been described as methylation biomarkers for prostate cancer, including the three genes that are the subject of this analysis. *GSTP1* is likely the most studied epigenetic lesion in relation to prostate cancer [26-28]. In addition, the gene is also of potential use as a screening marker in non-invasive samples such as urine and blood [29]. *APC* and *RASSF1* have also been described as prostate cancer markers with important roles in detecting the epigenetic field effect surrounding cancer foci [14,27]. In this paper, a multiplex assay detecting methylation of *GSTP1APC* and *RASSF1* has been developed and evaluated. The transition of singleplex to multiplex should lead to a decreased variance and restrain potential sources of error. In addition, it allows smaller sample quantities to be used as input for the assay, which is critical given that often limited cancer is found in needle biopsy tissue.

Multiplexing the three epigenetic assays and the *ACTB* control gene into one reaction leads to copy number and methylation ratio alterations relative to individual singleplex assays. The use of different fluorophores for the individual primers is most likely the main reason, although altered thermodynamics and PCR efficiencies cannot be completely excluded. The effect appears to be consistent for each individual assay, but not between different assays (Figure 1). Although this changes the interpretation of the calculated methylation ratios, the final outcome, i.e. detection or absence of methylation, should be determined with the same accuracy as with individual singleplex assays. Although only a small test cohort was available for verification, similar correlations between methylation and the presence of cancer foci were obtained. A small decrease in MCC was observed for *APC* and *RASSF1*. However, it can be argued that due to the consistency in copy number changes, the ratios should also be altered in a consistent manner. This supports the belief that the observed, minor decreases in correlation are random effects, errors that are not linked to the transition of singleplex to multiplex.

A typical biopsy tissue core using an 18-gauge needle produces a cylindrical mass of approximately 1 mm thick. A variable amount of tissue is cut from the FFPE block for analysis, especially if an atypical focus is noted and recuts are performed. This leaves an uneven amount of tissue remaining within the paraffin block. Thus, the smaller the tissue requirement to do further testing, including analytical, molecular analyses, the better.

The assays were initially developed using prostatectomy samples and 40 microns of biopsy tissue from FFPE blocks. However, such amounts of tissue are not always readily available for confirmatory assays, e.g. confirming a negative histology outcome or detecting occult cancer. Therefore, it was tested whether the multiplex assay could generate the same methylation outcome in smaller biopsies of 20 or even 10 μm. The smallest biopsy volume clearly possessed a suboptimal performance, where methylation was more often missed due to erroneous sample localization (Table 1). The minimally required amount of input DNA for reliable, repeatable detection of methylation was not observed for the 10 μm, as opposed to 20 and 40 μm samples (Figure 2 and Table 1). The occasional lack of detecting methylation in cancer-positive samples from 20 μm biopsies could relate to tissue sampling errors, older samples or lack of sufficient input. Additionally, not all tissue sections may have methylated promoter regions for any of the 3 genes. In general, the intuitive rule that more recent, larger samples are better for Q-MSP analysis was proven true (Table 1). Because 20-micron samples preserved in FFPE for up to 5 or 6 years seem to be borderline, requiring more DNA for testing seems reasonable for older, archived samples. As a general guideline, samples down to 20 micron provide sufficient input DNA up to an age of 6 years. For recently archived tissue, up to one year, 20-micron samples appear as adequate as their 40-micron counterparts.

A 2-fold dilution experiment ranging from 512 to 2 copies of the plasmid vector used for the multiplex assay was set up with 24 repeats per dilution factor (data not shown) to determine the limit of detection and analytical sensitivity of the assay. As little as 16 methylated copies of *APC* and *GSTP1* and 32 copies of *RASSF1* could be detected reliably, i.e. in more than 95% of the samples. This illustrates the high analytical sensitive of an MSP-based epigenetic assay.

In conclusion, an epigenetic, confirmatory multiplex assay for detecting the presence of prostate cancer cells missed by histology has been further developed. It performed similar relative to the individual singleplex assays for the most important test trait, biomarker gene methylation status. Epigenetic testing can be executed in an accurate and reliable manner in FFPE core tissue biopsies, with as little as 20 μm of sample volume.

## Methods

Thirty tissue samples for the multiplex versus singleplex experiment were obtained during routine prostatectomy as the standard care for 7 patients at the Institut de Pathologie Génétique (IPG, Gosselies, Belgium) and approved by the ethical committees of IPG Gosselies and CHU (Sart-Tilman, Belgium). Since this is a non-interventional, retrospective, subject-anonymized study, written patient consent was not required by the ethics committees. In addition, 12 cancer-negative BPH (benign prostatic hyperplasia) TURP (transurethral resection of the prostate) samples were obtained from a commercial supplier (Biona Center for Biotechnology, Cluj-Napoca, Romania).

Samples for the tissue minimization experiment were obtained from 51 patients, all with Gleason sum 6 (3 + 3) adenocarcinoma of the prostate, undergoing routine prostate biopsy procedures at The Johns Hopkins Medical Center. Testing of these anonymized samples was approved by the IRB at the Johns Hopkins Medical Institutions. Two sets of recent and older specimens were compared, 27 patients’ samples were obtained in 2011, 16 in 2005 and 8 in 2006. For 46 patients multiple, up to 4, cores were available for testing, totaling to 102 unique biopsy samples to be analyzed. Whenever possible, seven 10 micron sections were taken from each of these FFPE blocks, and randomly divided as one tissue biopsy of 40 μm, one of 20 μm and one of 10 μm volume of test tissue.

Samples from all cohorts were prepared identically, i.e. cells were scraped from the biopsy slides, followed by deparaffinization (Deparaffinization Solution, Qiagen) and cell lysis (EpiTect Plus FFPE bisulfite kit, Qiagen). After the DNA was isolated, it was bisulfite treated (EpiTect Plus FFPE bisulfite kit, Qiagen), and split into two equivalent replicates for real-time Q-MSP (quantitative methylation specific PCR) analysis (QuantiTect Multiplex NoROX kit, Qiagen).

Epigenetic profiling of prostate biopsy samples was based on a modified, quantitative version of the original MSP protocol [30]. In short, a molecular-beacon-based approach was used to detect methylated copies of *GSTP1APC* and *RASSF1*, either as three separate reactions or multiplexed into one. For each reaction, *ACTB* was used as a control (Additional file 1: Table S1).

A standard curve was included in each run for quantitative purposes. To this end, custom plasmid vectors were ordered (IDT, Iowa, USA) and a stock solution containing 800.000 copies per 5 μl was prepared. A serial 10-fold dilution was made down to 8 copies per 5 μl. All 6 standards were included in each PCR run. The vectors contained either the relevant fragment of one gene (singleplex reactions) or all relevant fragments of each one of the 4 genes (multiplex reactions). The standard curves allowed copy number calculations to be performed for each run. Normalized methylation ratios were calculated by dividing the detected amount of methylated copies by the amount of detected *ACTB* copies and multiplying this result by 1000 for interpretability.

All analyses were performed using R and BioConductor, including the ROCR package [31,32].

## Abbreviations

BPH, Benign prostatic hyperplasia; FFPE, Formalin-fixed paraffin-embedded; MCC, Matthews correlations coefficient; MSP, Methylation specific PCR; TURP, Transurethral resection of the prostate.

## Competing interests

This research project was funded by MDxHealth. LVN, JB, GO, JC & WVC are employees of or consulting for MDxHealth and may have corporate stock or stock options. MDxHealth has (pending) patents and exclusive rights on the assay and plans to launch an assay similar to the one described.

## Authors’ contributions

LVN drafted the manuscript, participated in the design of the study, performed bio-informatics and statistical analyses and interpreted the data. JB participated in the study design, project coordination, helped with data interpretation and to draft the manuscript. AT identified and provided protocol-specific and assisted with project coordination. GO carried out the molecular work, multiplex design and MSP sample testing. JC participated in the study design and project coordination. PD provided samples and participated in the study design. WVC assisted with analyses and data interpretation. JE conceived the study, participated in its design, provided samples and helped to draft the manuscript. All authors read, critically revised and approved the final manuscript.

## Pre-publication history

The pre-publication history for this paper can be accessed here:

http://www.biomedcentral.com/1471-2490/12/16/prepub

## Supplementary Material

Additional file 1 **VanNesteLetal_Supplementary_BMCUrol_rev1 contains Additional file****1****:Table S1 (primers and beacons) and Additional file ****1****:Figure S1 (illustrative ROC curves).** Click here for file
