# Supplementary material for: A tissue biopsy-based epigenetic multiplex PCR assay for prostate cancer detection
Source: BMC Urol. 2012 Jun 6;12:16. doi: 10.1186/1471-2490-12-16 (PMC3431995; doi:10.1186/1471-2490-12-16)
Supplement: Additional file 1 — VanNesteLetal_Supplementary_BMCUrol_rev1 contains Additional file1:Table S1 (primers and beacons) and Additional file 1:Figure S1 (illustrative ROC curves). [file 1471-2490-12-16-S1.docx]

**Supplementary Table 1**

| **Gene** | **Chromosome** | **Locus Start** | **Locus End** | **5' Beacon Modification^*^** | **3' Beacon Modification** |
| --- | --- | --- | --- | --- | --- |
| ACTB | 7 | 5571799 | 5571902 | CY5 | BHQ3 |
| GSTP1 | 11 | 67351144 | 67351235 | FAM | BHQ1 |
| APC | 5 | 112073458 | 112073548 | Texas Red | BHQ2 |
| RASSF1 | 3 | 50378153 | 50378289 | JOE | BHQ1 |

^*^ FAM was used as fluorophore for all singleplex reactions

**Supplementary Figure 1**

**
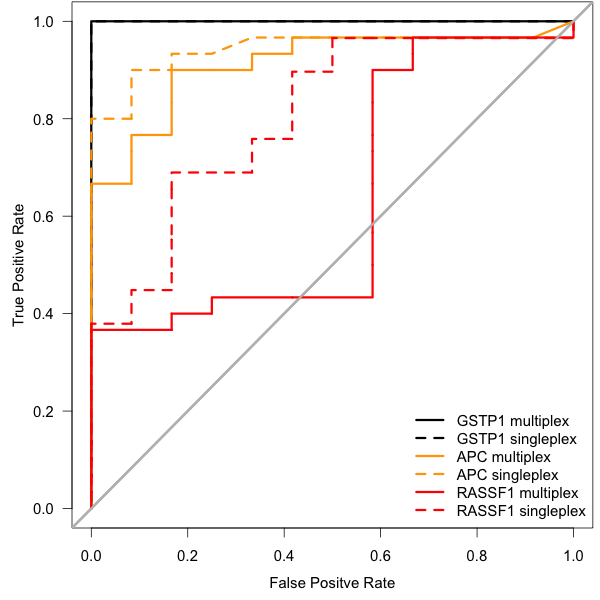
**

**Figure S1:** ROC curves for *GSTP1* (black), *APC* (orange) and *RASSF1* (red) for the singleplex (dashed lines) and multiplex (solid lines) assays. These plots are only generated for illustrative purposes, i.e. to show the technical feasibility of a multiplex assay. Two outliers are responsible for the suboptimal behavior of the *RASSF1* curve, similar to what is seen for the MCC in figure 1F. Strong conclusions cannot be made due to the low sample size. For this particular sample set, *GSTP1* would result in the ‘perfect’ molecular assay with a 100% sensitivity and specificity for low thresholds on the ratio.
